# Immune predictors of oral poliovirus vaccine immunogenicity among infants in South India

**DOI:** 10.1038/s41541-020-0178-5

**Published:** 2020-03-23

**Authors:** Sudhir Babji, Punithavathy Manickavasagam, Yin-Huai Chen, Nithya Jeyavelu, Nisha Vincy Jose, Ira Praharaj, Chanduni Syed, Saravanakumar Puthupalayam Kaliappan, Jacob John, Sidhartha Giri, Srinivasan Venugopal, Beate Kampmann, Edward P. K. Parker, Miren Iturriza-Gómara, Gagandeep Kang, Nicholas C. Grassly, Holm H. Uhlig

**Affiliations:** 1 Division of Gastrointestinal Sciences, Christian Medical College, Vellore, Tamil Nadu 632004 India; 2 Translational Gastroenterology Unit, Nuffield Department of Medicine, and Department of Paediatrics, University of Oxford, Oxford, OX3 9DU UK; 3 Department of Community Health, Christian Medical College, Vellore, Tamil Nadu 632004 India; 4 The Vaccine Centre, London School of Hygiene and Tropical Medicine, London, WC1E 7HT UK; 5 Institute of Infection and Global Health, University of Liverpool, Liverpool, L69 7BE UK; 6 Department of Infectious Disease Epidemiology, Imperial College London, London, W2 1PG UK

**Keywords:** Live attenuated vaccines, Paediatric research

## Abstract

Identification of the causes of poor oral vaccine immunogenicity in low-income countries might lead to more effective vaccines. We measured mucosal and systemic immune parameters at the time of vaccination with oral poliovirus vaccine (OPV) in 292 Indian infants aged 6–11 months, including plasma cytokines, leukocyte counts, fecal biomarkers of environmental enteropathy and peripheral blood T-cell phenotype, focused on gut-homing regulatory CD4+ populations. We did not find a distinct immune phenotype associated with OPV immunogenicity, although viral pathogens were more prevalent in stool at the time of immunization among infants who failed to seroconvert (63.9% vs. 45.6%, *p* = 0.002). Using a machine-learning approach, we could predict seroconversion a priori using immune parameters and infection status with a median 58% accuracy (cross-validation IQR: 50–69%) compared with 50% expected by chance. Better identification of immune predictors of OPV immunogenicity is likely to require sampling of mucosal tissue and improved oral poliovirus infection models.

## Background

The composition of the intestinal microbiota develops over the first few years of life, and is shaped by multiple factors such as mode of birth, breast-feeding, food intake and antibiotic use^1,2^. It is regulated by infant immunity, which in turn develops and is shaped by exposure to the microbiota^3,4^. Intestinal lymphoid tissues require the intestinal microbiota for normal development, and exposure to commensal and pathogenic organisms sets the tone of the systemic and mucosal immune system for the long term. These early interactions have a substantial impact on the development of metabolic and immune-mediated disorders^5^. There is also emerging evidence in mice and humans for microbiota dependent modeling of local immunity and the subsequent response to infection or vaccination^6–8^. Understanding the complex interaction between the microbiota and immune responses during infancy therefore has potentially important implications for vaccine development as well as prevention of immune-mediated disorders.

Infants in low and lower-middle income countries (LMICs) are exposed to a high burden of intestinal pathogens from birth^9,10^. It is thought that these infections alter intestinal immune system homeostasis and eventually lead to environmental enteropathy (EE). This understudied disorder is commonly found in children in the poorest socioeconomic settings and is characterized by intestinal inflammation, permeability and histological changes including blunted intestinal villi^11^. Recent work has begun to elucidate its contribution to malnutrition and changes in the microbiota that may adversely affect nutrient uptake and growth^12–14^.

It has also been suggested that EE may cause poor oral vaccine immunogenicity^15,16^. Oral vaccines against poliovirus, rotavirus, and cholera are all substantially less immunogenic and effective when given to children in LMICs^17^. This phenomenon has prolonged polio eradication by limiting vaccine effectiveness^18^, and is limiting the benefits from the global scale-up in access to oral rotavirus vaccines^19^. However, recent studies of biomarkers of EE in children at the time of immunization have had mixed findings, with different biomarkers showing positive, negative or no association with oral poliovirus or rotavirus vaccine immunogenicity^20^. Several other mechanisms may also contribute to poor oral vaccine immunogenicity in LMICs, including interference by high titers of antigen-specific transplacental or breastmilk antibodies. However, withholding breastfeeding does not improve oral poliovirus or rotavirus vaccine response^21–24^, and neonatal immunization when transplacental antibody titres are at their highest has in general resulted in comparable levels of seroconversion to doses administered later in life^25,26^.

In the case of oral poliovirus vaccine (OPV), enterovirus infection at the time of vaccination is associated with a modest decline in vaccine immunogenicity^27,28^. Recent data suggest this may be the case for oral rotavirus vaccine too^29^, which is also affected by co-administration of the live-attenuated OPV^30^. The mechanisms underlying this association are unclear and may relate to direct interference at mucosal and epithelial sites affecting virus replication and modulation of innate and adaptive immunity.

Here we report an investigation of the mechanisms behind poor OPV immunogenicity and the contribution of changes in systemic and mucosal immune homeostasis, including EE. We measured systemic and mucosal immune parameters in peripheral blood and stool samples collected at the time of monovalent serotype 3 OPV immunization of seronegative Indian infants aged 6–11 months. These infants were enrolled in a randomized controlled trial of the effect of the antibiotic azithromycin on EE and OPV immunogenicity^31^. As primary outcome, we reported that azithromycin treatment had no effect on seroconversion after OPV, despite reducing the enteric bacterial pathogen load and fecal biomarkers of EE. Instead, infection with enteric viruses (enteroviruses and rotavirus) was associated with seroconversion. We now report on extensive immune profiling of these infants at the time of immunization, including mucosal homing immune cells in peripheral blood, plasma cytokines and acute-phase proteins, leukocyte counts, and fecal biomarkers of inflammation. We use a statistical learning approach to identify immune markers measured at the time of vaccination that predict OPV immunogenicity. We end with a consideration of the implications of our findings for improving oral vaccines and oral vaccination strategies.

## Results

### Study population

**Fig. 1:**
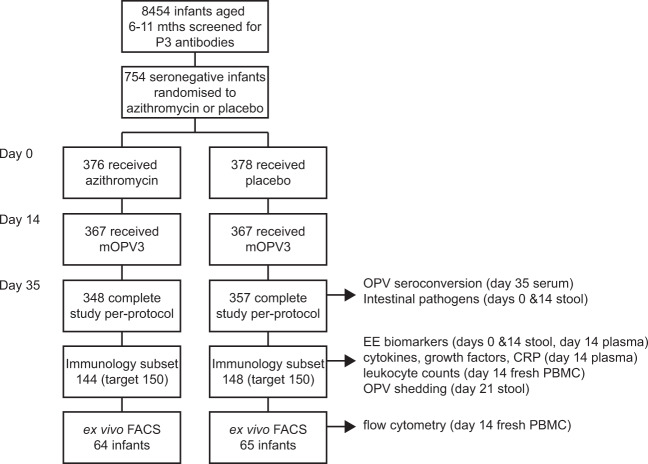
Flow chart of infants included in the analysis. P3 poliovirus serotype 3, mOPV3 monovalent serotype 3 oral poliovirus vaccine, PBMC peripheral blood mononuclear cells, EE environmental enteropathy, CRP C-reactive protein.

We measured markers of innate and adaptive, mucosal and systemic immunity in Indian infants aged 6–11 months and who lacked detectable antibodies at 1:8 dilution to serotype 3 poliovirus at the time of immunization with monovalent type 3 OPV (mOPV3) as part of a clinical trial of the effect of azithromycin on the immunogenicity of this vaccine. In the original study, 50% of infants given azithromycin seroconverted after vaccination with mOPV3, compared with 54% given placebo. Out of the 300 infants randomly selected for this study (stratified by study arm and seroconversion status), 292 completed the study per protocol and were included in our analysis of immune markers. Ex vivo flow cytometry was performed in 129 infants to examine circulating gut-homing T-cell phenotypes (Fig. 1).

### Immune measurements

Total cell counts were measured from freshly collected blood samples. We separated peripheral blood mononuclear cells (PBMCs) and plasma within 4–6 h of collection and measured lymphocyte gut-homing markers, plasma cytokines and acute phase proteins (see Methods section for full details). Data on seroconversion to mOPV3, vaccine virus shedding 7 days after immunization, stool inflammatory biomarkers and pathogens detected by Taqman array card (TAC) were also analyzed as previously reported^31^.

**Fig. 2:**
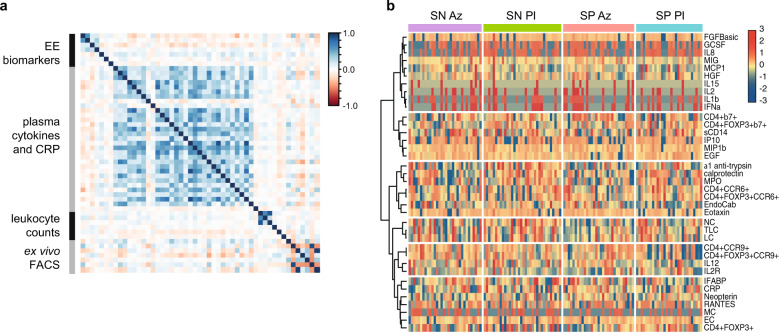
Correlation and clustering analysis of infant immune status variables. **a** Pearson’s correlation coefficient for the 51 variables in each of the 4 measurement “modules”. Pairwise comparisons are based on all available data (*n* = 292 infants for data on biomarkers of environmental enteropathy (EE), plasma cytokines and CRP, and leukocyte counts; and *n* = 129 infants for data on ex vivo flow cytometry). **b** Heatmap for each infant with variables (rows) clustered using Ward’s minimum variance hierarchical clustering method and infants (columns) grouped by treatment arm and OPV seroconversion status. A tree with 6 clusters showing the grouping of the variables is shown on the left of the heatmap and the variables included in each group on the right. In both plots the variables were log-transformed, normalized, and truncated at +/−3 standard deviations before analysis and plotting. Color bars indicate the scale for each plot. Only infants with complete data for all variables were included and invariant variables after normalisation and truncation were removed (126 infants, 37 variables). SN seroconversion negative, SP seroconversion positive, Az azithromycin arm, Pl placebo arm.

A cross-correlation matrix among all investigated markers shows strong associations among parameters within each class of measurements (measurement ‘module’) but weaker associations across modules (Fig. 2a). Unsupervised clustering of immune parameters did not reveal any strong association of immune phenotype with seroconversion to mOPV3 or treatment arm (Fig. 2b).

### Demographic and seasonal correlates of immune phenotype

Fecal calprotectin and other biomarkers of EE, C-reactive protein (CRP) as a measure of systemic inflammation, plasma cytokines and leukocyte populations, did not differ significantly by age-group or sex after false discovery rate (FDR) correction of *p*-values (Table 1). There were, however, significant differences in measurements according to the time of year the sample was collected, suggesting an important seasonal component. Measures of intestinal inflammation, including calprotectin, were raised among infants immunized in September–October compared with November–December (Table 1). In contrast, markers of systemic inflammation including interferon-γ (IFN-γ) and interleukin-1β (IL-1β) in plasma and neutrophil counts, were higher during November–December. Nearly all infants (271/292) were breastfed and breastfeeding status was not associated with immune phenotype (*p* > 0.8 for all variables in Table 1).

**Table 1:** Variables describing immune status by infant characteristics.

Infant characteristics (*n*)	Calprotectin (μg/g)	EE^a^ (%)	CRP (mg/l)	IFN-γ (pg/ml)	IL-1β (pg/ml)	CD4+FOXP3+ B7+ (cells/μl)	CD4+FOXP3+ CCR9+ (cells/μl)	Neutrophil count (cells/μl)
Seroconversion to OPV								
No (145)	1043.36 (66.87)	52.8 (76/144)	0.95 (0.08)	7.37 (2.97)	29.25 (4.46)	52.63 (0.92)	16.43 (0.97)	3653.03 (168.28)
Yes (147)	918.17 (60.89)	45.6 (67/147)	0.87 (0.07)	2.62 (1.09)	21.45 (3.39)	53.32 (1.23)	13.52 (1.11)	3397.31 (141.18)
*p*-value	0.548	0.587	0.874	0.548	0.483	0.753	0.483	0.587
Shedding of OPV								
No (135)	1082.46 (68.32)	54.8 (74/135)	0.95 (0.09)	5.51 (2.61)	25.73 (4.22)	53.2 (0.95)	16.14 (1.02)	3667.31 (180.75)
Yes (157)	891.56 (59.54)	44.2 (69/156)	0.87 (0.06)	4.52 (1.89)	24.98 (3.75)	52.73 (1.16)	14.08 (1.06)	3401.32 (132.14)
*p*-value	0.536	0.638	0.953	0.953	0.953	0.967	0.812	0.812
Study arm								
Azithromycin (144)	862.18 (65.47)	48.3 (69/143)	0.88 (0.06)	2.07 (0.94)	28.95 (4.38)	51.75 (0.93)	16.35 (1.03)	3348.52 (152.2)
Placebo (148)	1094.07 (61.34)	50 (74/148)	0.94 (0.08)	7.81 (2.96)	21.8 (3.51)	54.14 (1.17)	13.77 (1.05)	3695.32 (157.26)
*p*-value	**0.022**	0.901	0.981	0.384	0.507	0.204	0.461	0.204
Age (months)								
6–7 (195)	968.12 (53.89)	45.4 (88/194)	0.91 (0.07)	5.47 (2.25)	28.27 (3.95)	52.71 (0.99)	15.84 (0.92)	3678.22 (142.26)
8–11 (97)	1004.11 (82.9)	56.7 (55/97)	0.9 (0.07)	3.99 (1.48)	19.41 (2.74)	53.51 (1.04)	13.24 (1.18)	3214.88 (161.98)
*p*-value	0.986	0.283	0.533	0.706	0.836	0.986	0.374	0.283
Sex								
F (155)	975.09 (60.69)	49.7 (77/155)	0.93 (0.07)	3.45 (1.75)	23.44 (3.94)	53.29 (1.07)	14.72 (1.01)	3540.6 (144.47)
M (137)	985.85 (67.99)	48.5 (66/136)	0.88 (0.07)	6.71 (2.72)	27.46 (3.97)	52.56 (1.06)	15.44 (1.1)	3505.85 (167.94)
*p*-value	0.955	0.955	0.902	0.863	0.863	0.902	0.955	0.902
Time of year								
Sep–Oct (121)	1108.95 (65.84)	52.9 (64/121)	0.94 (0.08)	0.45 (0.45)	10.76 (3.26)	52.08 (2.08)	13.47 (1.35)	3106.61 (135.26)
Nov–Dec (171)	888.42 (60.86)	46.5 (79/170)	0.88 (0.07)	8.18 (2.65)	35.63 (4.01)	53.23 (0.75)	15.55 (0.88)	3819.85 (157.61)
*p*-value	**0.003**	0.332	0.774	**0.005**	**<0.001**	0.745	0.288	**0.007**
Number of bacterial pathogens in stool								
0 (65)	877.98 (92.59)	46.2 (30/65)	0.84 (0.09)	7.6 (5.18)	27.05 (7.51)	52.96 (1.2)	16.08 (1.43)	3094.2 (202.53)
1 (89)	924.09 (82.24)	55.1 (49/89)	0.96 (0.1)	2.94 (1.52)	24.24 (4.33)	53.6 (1.22)	15.88 (1.32)	3625.62 (217.36)
>1 (137)	1064.98 (66.43)	46.7 (64/137)	0.9 (0.08)	5.1 (2.08)	25.01 (3.9)	52.64 (1.27)	14.22 (1.14)	3667.17 (158.32)
*p*-value	0.986	0.986	0.986	0.986	0.986	0.986	0.986	0.986
Number of viral pathogens in stool								
0 (132)	912.98 (65.49)	50.8 (67/132)	0.86 (0.07)	4.23 (2.11)	23.55 (3.91)	53.54 (0.97)	15.42 (1.16)	3362.96 (155.15)
1 (117)	1086.58 (77.33)	47.9 (56/117)	1.03 (0.1)	5.35 (2.99)	27.95 (5.22)	51.27 (1.36)	14.51 (1.08)	3675.99 (181.15)
>1 (42)	894.56 (94.09)	47.6 (20/42)	0.7 (0.1)	6.41 (2.73)	22.94 (4.11)	56.27 (1.53)	15.92 (2.2)	3623.88 (300.25)
*p*-value	0.986	0.986	0.986	0.986	0.986	0.745	0.986	0.986
Enterovirus in stool								
No (180)	967.08 (57.29)	53.3 (96/180)	0.9 (0.07)	6.88 (2.48)	24.55 (3.48)	53.82 (0.84)	16.29 (0.96)	3437.29 (137.68)
Yes (111)	1001.27 (74.06)	42.3 (47/111)	0.91 (0.08)	1.93 (0.95)	26.34 (4.76)	51.96 (1.35)	13.63 (1.15)	3671.1 (183)
*p*-value	0.933	0.927	0.933	0.927	0.935	0.927	0.927	0.927

Seven variables are shown from a total of 51 that were measured in this analysis and also a composite indicator of EE (see Supplementary Table 1 for full list of variables compared with seroconversion/OPV shedding). Table entries show mean (standard error) or proportion as a percentage.

^a^Presence of EE yes/no based on whether the infant has at least 1 biomarker of EE in the top 10th percentile; *p*-values are FDR corrected, shown in bold if <0.05 and italics if <0.1.

### Effect of intestinal infection with pathogenic viruses and bacteria

The number of bacterial or viral pathogens detected in stool at the time of vaccination did not show any significant correlation with EE biomarkers, plasma cytokines, CRP or lymphocyte populations after FDR correction of *p*-values (Table 1). Calprotectin was raised in infants with bacterial pathogens detected in stool, in agreement with expectations from previous work, but this was not significant after FDR correction^31^. Viral pathogens were more prevalent among those infants who failed to seroconvert to OPV (prevalence of at least one viral pathogen was 63.9% vs. 45.6%, Fisher’s *p* = 0.002), as previously reported^31^. However, detection of any pathogenic virus in stool was not correlated with any of the immune parameters after FDR correction.

### Effect of treatment with azithromycin

As reported in Grassly et al.^31^, treatment with azithromycin reduced fecal biomarkers of EE (myeloperoxidase, calprotectin, and α1-antitrypsin) and the prevalence of pathogenic *Escherichia coli* and *Campylobacter* bacteria detected in stool, but did not affect seroconversion to OPV which was 50% and 54% in the treatment and placebo arms respectively. Here we report that azithromycin, despite significantly reducing fecal calprotectin level, did not affect measures of systemic inflammation such as CRP (0.88 mg/L among infants in the treatment arm at the time of vaccination compared with 0.94 mg/L in the placebo arm) or other immune parameters of interest, including circulating CD4+ T cells expressing intestinal or mucosal homing markers and the regulatory cell marker forkhead box P3 (FOXP3) (Table 1).

### Association with OPV seroconversion and vaccine shedding

After FDR correction none of the 51 immune parameters showed a significant individual association with OPV seroconversion or shedding of vaccine virus as a marker of vaccine “take” (Table 1 and Supplementary Table 1). Measures of mucosal inflammation (e.g., fecal calprotectin, myeloperoxidase) and systemic inflammation (e.g., plasma IFN-γ and IL-1β) measured in plasma at the time of vaccination were not significantly different among infants according to their subsequent seroconversion or vaccine shedding status (Table 1 and Supplementary Fig. 1). The number of regulatory CD4+ T cells homing to the small intestine (CCR9+) was higher in infants who failed to seroconvert, but this was not significant after FDR correction (16.4 vs. 13.5 cells/μl, FDR *p*-value = 0.483).

### Statistical learning analysis

**Fig. 3:**
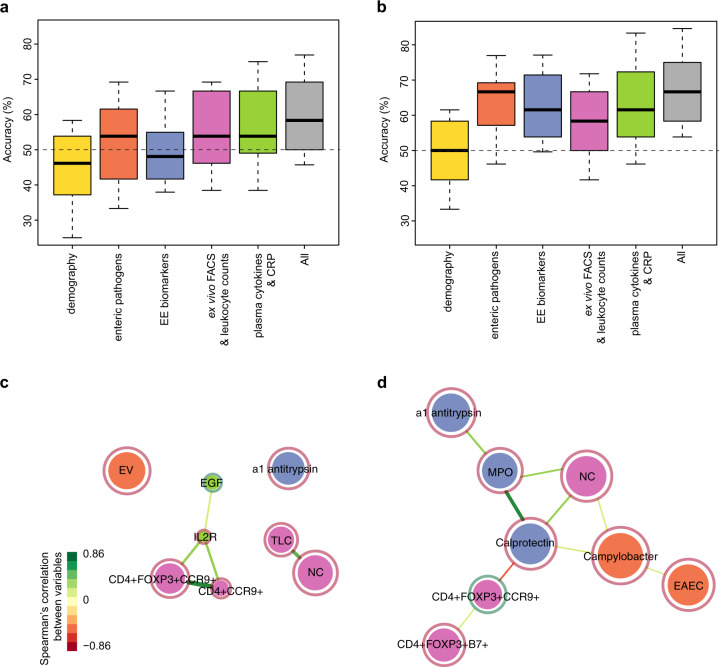
Random forests analysis to predict seroconversion and study arm. The accuracy of random forests analysis to predict **a** seroconversion and **b** study arm for each measurement module individually and all modules combined. For each analysis we performed a 10-fold cross-validation repeated 20 times. The boxes correspond to the interquartile range for the accuracies in the prediction set, with the solid line showing the median and the whiskers extending to the 10th and 90th percentile. The dashed line indicates expected accuracy if a random choice were made. The top eight most important variables that predict **c** seroconversion and **d** study arm in one best fit full random forests model are shown as correlation networks. The color of the circle around each variable indicate whether they were positively (green) or negatively (red) associated with seroconversion or azithromycin respectively. The size of each network node indicates the strength of correlation with the outcome and the color indicates whether the variable describes pathogens in stool (orange), EE biomarkers (blue) ex vivo T-cell and total leukocyte count data (pink), or plasma cytokines (green) (as for **a** and **b**). Demographic variables were not among the top eight variables. Lines connect circles with a Spearman correlation coefficient of at least 0.2, with the color of the line indicating the strength of the correlation (indicated by color scale bar). EV enterovirus, EGF epidermal growth factor, IL2R interleukin-2 receptor, TLC total leukocyte count, NC neutrophil count, MPO fecal myeloperoxidase, EAEC enteroaggregative *Escherichia coli*. Analysis is for infants with complete data for all variables only (*n* = 126 infants).

We used supervised learning (random forest) analysis of immune measurements taken at the time of vaccination to determine whether they allowed accurate out-of-sample prediction of infant response to OPV or receipt of azithromycin. We analyzed the accuracy of each module independently and combined using 10-fold cross-validation and plotted the correlation network for the eight most important variables in a single run of the random forest algorithm for the combined analyses (Fig. 3).

Combining all measurement modules for 126 infants with complete data we were able to predict seroconversion with a median accuracy across all cross-validation samples of 58% (interquartile range (IQR): 50–69%; Fig. 3a). This compares with 50% accuracy expected by random choice assuming a priori equal probabilities of seroconversion or not. The most important measurement modules contributing to this modest predictive accuracy were enteric pathogens (enterovirus in stool), ex vivo flow cytometry measurements (regulatory CD4+ T cells expressing CCR9), neutrophil and the (correlated) total leukocyte count and plasma cytokines (Fig. 3c). The out-of-sample predictive accuracy for the combined modules to predict shedding of poliovirus after immunization was not different to that expected by chance (Supplementary Fig. 2).

Infant study arm (receipt of azithromycin or placebo 2 weeks before sample collection at vaccination) was predicted with a median accuracy of 66.7% (IQR: 58.3–75.0%; Fig. 3b). All measurement modules contributed to this predictive accuracy with the exception of demography (as expected because of the randomized study design). Important variables included measures of intestinal inflammation and integrity (calprotectin, myeloperoxidase, α1-antitrypsin), regulatory T cells homing to the intestine and enteric bacterial pathogens (*Campylobacter*, enteroaggregative *Escherichia coli*) (Fig. 3d).

## Discussion

Our study provides insight into the ecology of the gastrointestinal tract and the developing immune system in infants in a low-income setting with a high load of bacterial and viral intestinal pathogens. Perturbing the intestinal microbiota with azithromycin allowed us to overcome some of the difficulties with complex association data in observational studies and reveals distinct bacteria-associated changes in infant immune status. To analyze these complex data including over 30 infection and 50 immune parameters per child we used a systems biology approach to identify immune signatures that could predict OPV immunogenicity.

We did not find a distinct immune phenotype that was strongly associated with OPV seroconversion or shedding among the immune parameters that we measured. As previously reported, infection with enteroviruses at the time of vaccination was more common among infants who failed to seroconvert to OPV^31^, but these infections were not associated with a distinct immune signature in peripheral blood or stool (Fig. 2b). Combining all measurements in a machine-learning (random forests) analysis, OPV seroconversion could be predicted with an out-of-sample accuracy of 58% (IQR: 50–69%), only a modest improvement over the 50% accuracy expected by chance. Apart from enterovirus infection, the variables with the highest importance in this analysis were the numbers of CD4+ T cells homing to the small intestine (CCR9+), particularly CD4^+^FOXP3^+^ (regulatory) T cells, and also circulating leukocytes, in particular neutrophils, and the fecal biomarker of enteropathy α1-antitrypsin. This is potentially suggestive of a local intestinal inflammation that inhibits OPV immunogenicity. CD4+ regulatory T cells have a well-established role in intestinal inflammation—they home towards the inflamed gut and can resolve intestinal inflammation^32^. They are also involved in the early antiviral immune response to mucosal infection in mice^33,34^. However, without mucosal tissue samples, it is difficult to conclude their role in the response to OPV in these infants.

Shedding of poliovirus after vaccination was strongly correlated with seroconversion, and markers of inflammation were somewhat raised among infants who did not shed poliovirus after vaccination as seen for seroconversion (although these differences were not significant in univariate analyses after FDR correction). However, shedding of OPV after immunization was not predicted with any degree of accuracy above that expected by chance on the basis of the immune parameters that we measured.

We did find significant seasonal variation in several immune parameters, including raised measures of intestinal inflammation and lower levels of systemic proinflammatory plasma cytokines (IL-1β, IFN-γ) and neutrophil counts in the warmer months of September to October compared with November to December. This may reflect differences in exposure to infection, with many diarrheal pathogens more common in the warmer months in this population and respiratory illness peaking in colder months^9,35^.

Treatment of infants with azithromycin resulted in a network of correlated immune changes that were distinct from those associated with OPV seroconversion. This is consistent with the absence of an effect of treatment on vaccine response. Prior receipt of azithromycin or placebo could be predicted with a median of 67% out-of-sample accuracy (IQR 58–75%). In addition to the previously reported reduction in bacterial pathogens and biomarkers of intestinal inflammation, important variables in the random forests analysis included gut-homing (β7+ or CCR9+) regulatory CD4+ T cells and neutrophil counts. Systemic inflammation measured by CRP in plasma was not affected by treatment, despite the known anti-inflammatory effects of azithromycin, which may be mediated both through a reduction in bacterial load and direct effects on cell signaling pathways^36^.

Our study has a number of limitations. It is observational in nature and lacks mucosal tissue that would have allowed a more direct assessment of intestinal immunity. Although pediatric endoscopy has an excellent safety record, we decided not to take intestinal biopsies because infants were asymptomatic and healthy despite raised levels of EE biomarkers. We therefore relied on inference from the systemic compartment and from circulating cells expressing mucosal and gut-homing receptors/integrins. Further investigation of underlying mechanisms responsible for oral vaccine failure may benefit from examination of tissue biopsies taken during diagnostic procedures and use of appropriate in vitro and animal models^37,38^.

An additional limitation is that we focused on a single population of seronegative infants in south India with high levels of EE biomarkers. Indeed, fecal biomarkers of inflammation in our study are among the highest reported from any cohort described so far in the world^39^. It will be important to determine how our findings in this population translate to other settings, such as in sub-Saharan Africa, where oral vaccine immunogenicity is also compromised. We deliberately enrolled infants without detectable serum neutralizing antibodies to serotype 3 poliovirus, but these infants were previously exposed to trivalent oral poliovirus vaccine and may have developed an immune response that was not detectable in blood or that had waned by the time of enrollment. It is therefore possible that our population included both naïve and immunologically primed infants with a potential impact on the subsequent immunogenicity of OPV. However, we were not able to distinguish such a population and analysis of infant vaccination history and seroprevalence at screening for eligibility was consistent with a simple hypothesis of a small, independent probability of vaccine failure per dose of earlier vaccine^40^.

Finally, we do not report on the commensal intestinal microbiota here, which is known to have immunomodulatory effects^3^. However, in a subset of infants from this study the bacterial microbiota was assessed by 16S rRNA sequencing of stool and found not to correlate with OPV immunogenicity or shedding^28^.

In conclusion, our study did not find a strong predictor of OPV immunogenicity in this population in south India. Infection with enteric viruses and changes in gut-homing regulatory T cells showed a modest association with OPV immunogenicity. Further work would be required to investigate this possible association, ideally including mucosal samples. It is clear that intestinal homeostasis among these infants is skewed towards an inflammatory state that maintains health in the presence of multiple intestinal pathogens. On average, each infant had >2 pathogen targets detected in stool using TAC but only 2% had diarrhea at enrollment^31^. We do not fully understand the mechanisms responsible for the development and maintenance of this inflammatory environment, although they are likely to reflect the integration by the infant immune system of multiple signals from intestinal viral and bacterial pathogens and commensals^3,41^. Further investigation of these mechanisms in humans and in animal models may identify pathways that could be targeted by coadministered drugs or adjuvants that enhance the immune response to oral vaccines.

Translating our current findings to interventions to improve oral vaccine immunogenicity and effectiveness is difficult. Treatment of enteric virus infection or directly perturbing infant intestinal immune environment from an inflammatory state that might be adaptive in this community may well be detrimental. However, alternative approaches could include changes to an earlier immunization schedule, including neonatal dosing, before exposure to pathogenic enteric viruses, or interventions to promote gut health of neonates, such as maternal immunization, water and sanitation interventions or promotion of exclusive breastfeeding.

## Methods

### Study population

Infants aged 6–11 months living in Vellore, south India, were enrolled in a double-blind, randomized, placebo-controlled trial to study the effect of a 3-day course of azithromycin on serotype-3 poliovirus vaccine immune response. Infants were enrolled in the study if they lacked detectable antibodies against serotype 3 poliovirus at a dilution of 1 in 8 and were medically fit. They were excluded if they had a history of allergic reaction after oral poliovirus vaccine, had chronic diarrhea (>14 days), were receiving immunosuppressant medication, or they or their mother had syndromic or documented evidence of being immunocompromised. Full details of the study population and the result of treatment with azithromycin have been published previously^31^. For the present study, we selected a random sample of 300 infants from those who had completed the trial and had sufficient sample volumes, with equal numbers chosen by study arm and poliovirus seroconversion status (defined as the detection of serotype-3 poliovirus-specific serum neutralizing antibodies at a dilution of 1 in 8 or higher in blood taken 21 days after vaccination).

The trial was performed in accordance with good clinical practice and ethical principles of the Declaration of Helsinki, including the collection of written informed consent from parents. The study was approved by the Institutional Review Board of the Christian Medical College Vellore and the Drugs Controller General of India. All biological samples were coded with a unique ID linked to the study participant ID, to ensure that only anonymized samples were analyzed and that laboratory personnel were blinded to study group assignment. The trial was registered with the Clinical Trials Registry India on 9 May 2014, number CTRI/2014/05/004588.

### Full blood count and isolation of peripheral blood mononuclear cells

Total white blood cell count and differential leukocyte count (lymphocytes, neutrophils, monocytes and eosinophils) were performed in blood collected in EDTA tubes based on standardized methods by Leishman’s staining in a Neubauer’s chamber by trained technicians using standardized methods. Peripheral blood mononuclear cells (PBMC) were isolated by Ficoll Hypaque gradient method on the day of vaccination (day 14 of the trial). PBMC were analyzed ex vivo using flow cytometry (FACS), within 4–6 h of collection.

### FACS analysis

Samples were acquired on a BD FACSAria™ III running a BD FACSDIVA Ver.8.0.1 software and results analyzed using FlowJo X 10.0.6 software (Supplementary Table 2). Cell viability was determined using LIVE/DEAD® Fixable Aqua Dead Cell Stain Kit. Appropriate single stained controls and florescent minus one (FMO) controls were used. The PBMCs collected on the day of administration of monovalent OPV3 (pre-vaccination), were assessed for the lymphocyte subsets with a special focus on the gut-homing subpopulations. Staining was performed for CD3, CD4, CCR6, CCR9, FOXP3, and β7-integrin (Supplementary Table 2). This allowed us to define the following populations: CD3^+^ T cells, CD3^+^CD4^+^ T cells, CD3^+^CD4^+^FOXP3^+^ regulatory T cells, and investigate CCR6+, CCR9+, and integrin β7+ gut homing subsets (gating strategy shown in Supplementary Fig. 3).

### Plasma cytokine and C-reactive protein levels

Plasma was separated from blood samples collected on the day of vaccination and stored at −80 °C until analysis. Samples were tested for a diverse range of pro-inflammatory and anti-inflammatory cytokines, growth markers and chemokines using the Human Cytokine Magnetic 30-plex panel and analyzed using a Luminex^TM^ platform. This includes chemokines (Eotaxin, IP-10, MCP-1, MIG, MIP-1α, MIP-1β, and RANTES), growth factors (EGF, FGF-basic, HGF, and VEGF), and cytokines (G-CSF, GM-CSF, IFN-α, IFN-γ, IL-1β, IL-1RA, IL-2, IL-4, IL-5, IL-6, IL-7, IL-8, IL-10, IL-12 (p40/p70), IL-13, IL-15, IL-17, and TNF-α). Results were calculated using the 5PL fit equation on the Bio-plex Manager software Version 6.1. Plasma C-reactive protein (CRP) levels were estimated using the ProcartaPlex Human CRP Simplex kit and analyzed using the Luminex^TM^ platform.

Plasma cytokine and CRP assays were run across multiple 96-well plates. We found significant plate-to-plate variation in estimated analyte levels using the Luminex^TM^ platform. This variation remained even after using more stringent thresholds for analyte minima (at value of 6th dilution standard across 7 dilutions) and checking readings across plates used to fit the standard curve were within 10% of each other. For example, the F-statistic for IL12 across the five plates used for plasma cytokine measurement was 22.2 (*p*-value < 0.001) and for CRP across the four plates used was 16.5 (*p* < 0.001). The distribution of samples across plates was approximately equal (plasma cytokines) or deliberately randomized (CRP) with respect to study arm (azithromycin vs. placebo) and outcome (seroconversion) and we therefore included these measurements in our analyses. However, this highlights the importance of checking batch effects in high-throughput studies.

### Environmental enteropathy biomarkers

Stool biomarkers of inflammation (myeloperoxidase, calprotectin), protein-losing enteropathy (α1-antitrypsin) and immune activation (neopterin), and plasma biomarkers of microbial translocation (soluble CD14 and endotoxin-core IgG) and epithelial damage (intestinal fatty acid binding protein) were measured on the day of vaccination using commercial enzyme-linked immunosorbent assays (ELISA)^31^. Stool aliquots for the fecal biomarker ELISA assays were supplemented with a cocktail of protease inhibitors before being stored at −70 °C.

### Detection of poliovirus shedding and stool pathogens

Shedding of serotype 3 Sabin poliovirus in stool samples collected 7 days after vaccination was assessed using a singleplex quantitative real-time PCR, using RNA extracted by Vx reagents on a Qiaxtractor^42^. Bacterial, viral, and eukaryotic pathogens were analyzed using total nucleic acid extracted from stool samples collected on the day of vaccination using an enteropathogen Taqman Array Card (TAC) quantitative PCR assay developed by the Division of Infectious Diseases and International Health, University of Virginia^31,43^. TAC assay targets allowed identification of pathogenic bacteria (*Aeromonas, Campylobacter, Clostridium difficile, Helicobacter pylori, Salmonella, Shigella, Vibrio cholerae*, and enteroaggregative, enteropathogenic, enterotoxigenic and shiga-toxin producing *Escherischia coli* (EAEC, EPEC, ETEC, and STEC)), viruses (Adenovirus, Astrovirus, Enterovirus, Norovirus, Rotavirus, and Sapovirus) and eukaryotes (*Ancylostoma, Ascaris, Cryptosporidium, Cyclospora, Enterocytozoon bieneusi, Entamoeba histolytica, Encephalitozoon intestinalis, Giardia, Isospora, Necator, Strongyloides, Trichuris*).

### Statistical analysis

Data on fecal and plasma biomarkers of environmental enteropathy, circulating ex vivo T-cell phenotype, plasma cytokines and leukocyte counts for samples collected on the day of vaccination (study day 14) were compiled in a single dataset. Correlation among the variables was assessed by calculating Pearson’s correlation coefficient for the log-transformed variables rescaled to have a mean of zero and standard deviation (SD) = 1. Univariable comparisons between groups were based on analysis of variance for the log-transformed variables or Wilcoxon’s (non-parametric) rank sum test for the untransformed data (two-sided tests). *p*-values for the univariable tests of significance were corrected for multiple comparisons using FDR correction^44^.

Hierarchical cluster analysis of variables in the complete dataset was performed using Ward’s minimum variance criterion^45^. Heatmaps of the clustered dataset were plotted to visualize the relationship between infant immune phenotype and study arm/seroconversion status. The association between the immune phenotype data and classification of infants according to their seroconversion status or study arm was assessed using the random forests algorithm^46^. For each analysis we report the median accuracy from a 10-fold cross-validation using 20 random forests for each fold. Variables were ranked by their importance in the random forests analysis and the top eight most important variables plotted in a correlation network with links between pairs of variables shown if their Spearman rank correlation coefficient was greater than 0.2. Variable importance was assessed by the mean decrease in the Gini coefficient resulting from their inclusion in the random forest model.

All analyses were performed in the R programming language (R Core Team. R: A Language and Environment for Statistical Computing. www.R-project.org). Individual R packages were used during the analysis including pheatmap for the heatmap plots, beeswarm for the univariable plots, randomForest for the random forest analyses, and igraph for the network plots.

### Reporting summary

Further information on research design is available in the Nature Research Reporting Summary linked to this article.

## Supplementary information

Supplementary Information

Reporting summary

## Data Availability

All laboratory data is available with the investigators and can be provided by the corresponding author upon reasonable request.
