# Supplementary material for: Immune predictors of oral poliovirus vaccine immunogenicity among infants in South India
Source: NPJ Vaccines. 2020 Mar 23;5:27. doi: 10.1038/s41541-020-0178-5 (PMC7089977; doi:10.1038/s41541-020-0178-5)
Supplement: Supplementary file 1 — Supplementary Information [file 41541_2020_178_MOESM1_ESM.pdf]

Supplementary Information:

Immune predictors of oral poliovirus vaccine immunogenicity among infants in south India

Sudhir Babji, Punithavathy Manickavasagam, Yin-Huai Chen, Nithya Jeyavelu, Nisha Vincy Jose, Ira Praharaj, Chanduni Syed, Saravanakumar Puthupalayam Kaliappan, Jacob John, Sidhartha Giri, Srinivasan Venugopal, Beate Kampmann, Edward PK Parker, Miren Iturriza-Gómara, Gagandeep Kang, Nicholas C Grassly, Holm H Uhlig

Supplementary Table 1 Comparison of *ex vivo* measurements according to whether infant seroconverted or shed poliovirus after administration of oral poliovirus vaccine.

Variable (units)	Seroconversion			Shedding		
	No	Yes	p-value	No	Yes	p-value
ex vivo flow cytometry and cell counts (all cells/µl)						
Leucocytes	13542.76 (341.42)	13542.18 (305.19)	0.854	13361.48 (355.94)	13698.09 (295.1)	0.762
Neutrophils	3653.03 (168.28)	3397.31 (141.18)	0.410	3667.31 (180.75)	3401.32 (132.14)	0.774
Lymphocytes	9282.99 (242.04)	9541.44 (215.26)	0.552	9124.25 (243.02)	9661.48 (215.11)	0.459
Eosinophils	497.41 (45.89)	514.05 (54.08)	0.406	462.1 (43.05)	543.55 (54.54)	0.430
Monocytes	87.2 (11.01)	89.16 (11.33)	0.879	85.13 (11.1)	90.82 (11.17)	0.963
Basophils	10.7 (3.29)	12.6 (3.55)	0.787	10.45 (3.39)	12.69 (3.43)	0.963
CD4+B7+	79.75 (0.67)	80.11 (0.81)	0.753	79.47 (0.71)	80.32 (0.75)	0.812
CD4+CCR6+	2.57 (0.15)	2.92 (0.23)	0.847	2.54 (0.16)	2.91 (0.21)	0.871
CD4+CCR9+	10.34 (0.75)	9.21 (0.8)	0.835	10.27 (0.82)	9.39 (0.73)	0.953
CD4+FOXP3+	2.75 (0.15)	2.62 (0.12)	0.885	2.9 (0.15)	2.5 (0.11)	0.638
CD4+FOXP3+B7+	52.63 (0.92)	53.32 (1.23)	0.753	53.2 (0.95)	52.73 (1.16)	0.967
CD4+FOXP3+CCR6+	10.42 (0.68)	11.43 (0.89)	0.885	9.95 (0.67)	11.75 (0.85)	0.812
CD4+FOXP3+CCR9+	16.43 (0.97)	13.52 (1.11)	0.483	16.14 (1.02)	14.08 (1.06)	0.812
EE biomarkers						
calprotectin (µg/g)	1043.36 (66.87)	918.17 (60.89)	0.340	1082.46 (68.32)	891.56 (59.54)	0.330
myeloperoxidase (ng/ml)	19723.76 (1445.17)	17424.91 (1275.59)	0.419	20000.73 (1524.53)	17317.85 (1215.88)	0.762
neopterin (nmol/l)	6710.46 (371.71)	6870.96 (444.21)	0.826	6805.53 (430.98)	6779.42 (392.33)	0.963
α1 anti-trypsin (mg/g)	1.28 (0.12)	1.02 (0.07)	0.273	1.27 (0.1)	1.05 (0.09)	0.423
soluble CD14 (ng/ml)	2787.7 (142.85)	2588.7 (131.88)	0.419	2716.48 (140.63)	2662.16 (134.59)	0.963
intestinal fatty acid binding protein (pg/ml)	863.28 (45.1)	770.25 (35.3)	0.273	872.93 (46.96)	767.88 (34.5)	0.430
endotoxin core antibody (MU/ml)	75.28 (12.91)	57.79 (9.5)	0.221	77.33 (13.83)	57.14 (8.93)	0.430
any biomarker in top 10 percentile (%)	52.8 (76/144)	45.6 (67/147)	0.400	54.8 (74/135)	44.2 (69/156)	0.430
plasma cytokines (all pg/ml)						
FGFBasic	35.32 (7.15)	31.69 (5.65)	0.483	31.53 (6.72)	35.19 (6.19)	0.967
IL1β	29.25 (4.46)	21.45 (3.39)	0.483	25.73 (4.22)	24.98 (3.75)	0.953
GCSF	343.36 (33.66)	281.07 (29.81)	0.483	321.05 (34.42)	304.22 (29.67)	0.953
IL10	28.36 (10.68)	23.73 (10.98)	0.847	22.09 (10.07)	29.42 (11.31)	0.953
IL13	40.03 (16.72)	26.8 (9.84)	0.895	27.24 (14.05)	38.63 (13.32)	0.899
IL6	6.22 (3.4)	1.61 (1.14)	0.483	5.89 (3.58)	2.18 (1.26)	0.812
IL12	335.08 (12.34)	303.2 (5.68)	0.483	326.87 (12.63)	312.29 (6.53)	0.932
RANTES	6370.23 (289.58)	7417.89 (1203.85)	0.861	6066.3 (309.67)	7612.5 (1125.52)	0.812
Eotaxin	31.66 (1.56)	25.94 (1.26)	0.242	31.21 (1.47)	26.7 (1.38)	0.536
IL17	15.01 (6.28)	11.07 (4.56)	0.895	9.95 (4.9)	15.67 (5.83)	0.953
MIP1α	10.33 (4.12)	0.54 (0.54)	0.242	8.19 (3.75)	3.01 (2.13)	0.697
GMCSF	4.59 (2.58)	5.03 (3.17)	0.861	3.24 (2.48)	6.16 (3.15)	0.812
MIP1β	135.15 (10.69)	126.72 (7.74)	0.861	131.06 (10.88)	130.78 (7.92)	0.953
MCP1	339.89 (37.51)	282.7 (22.42)	0.587	317.63 (34.03)	305.48 (28.18)	0.812
IL15	46.83 (17.08)	22.75 (9.46)	0.548	40.1 (16.84)	30.06 (10.92)	0.953
EGF	111.98 (5.83)	117.61 (6.84)	0.847	110.63 (6.48)	118.41 (6.24)	0.812
IL5	1.27 (0.78)	0 (0)	0.483	0.69 (0.52)	0.57 (0.57)	0.932
HGF	173.95 (27.94)	120.62 (5.77)	0.548	161.92 (28.12)	134.37 (10.8)	0.979
VEGF	3.98 (2.78)	0.6 (0.35)	0.753	3.58 (2.91)	1.17 (0.68)	0.963
IFNγ	7.37 (2.97)	2.62 (1.09)	0.548	5.51 (2.61)	4.52 (1.89)	0.953
IFNα	29.88 (6.16)	14.92 (2.27)	0.483	26.86 (6.33)	18.48 (2.77)	0.967
IL1RA	58.83 (19.72)	29.92 (14.33)	0.587	51.35 (19.38)	38.18 (15.36)	0.953
TNFα	3.36 (1.83)	0 (0)	0.483	2.51 (1.64)	0.95 (0.95)	0.812
IL2	14.04 (2.96)	11.86 (2.47)	0.847	12.53 (2.83)	13.3 (2.63)	0.967
IL7	5.1 (2.52)	0.84 (0.42)	0.757	2.71 (1.58)	3.16 (1.94)	0.953
IP10	54.45 (6.86)	34.3 (2.9)	0.483	54.13 (7.34)	35.87 (2.83)	0.812
IL2R	496.9 (68.32)	407.86 (19.58)	0.895	475.94 (70.82)	431.56 (25.07)	0.967
MIG	100.15 (7.86)	82.06 (3.8)	0.753	97.04 (7.25)	85.89 (5.2)	0.953
IL4	10.77 (6.23)	1.28 (1.28)	0.587	7.06 (4.98)	5.08 (4.05)	0.967
IL8	43.65 (5.36)	36.32 (4.13)	0.847	40.81 (5.43)	39.23 (4.21)	0.953
other biomarkers						
C-reactive protein (mg/l)	0.95 (0.08)	0.87 (0.07)	0.874	0.95 (0.09)	0.87 (0.06)	0.953

Data are mean (SE) or % (x/n). P-values are based on Wilcoxon rank sum test or Fisher's exact test respectively, corrected for multiple comparisons using the Benjamini and Hochberg false discovery rate algorithm.

Supplementary Table 2 Flow cytometry BD FACSAria™ III parameters used to characterize peripheral blood mononuclear cells

Laser Name	Wavelength (nm)	Detector Array	Mirror	Filter	Parameter	Antibody	Source (ID)	Volume/100ul reaction (ul)
Blue	488	Octagon	735 LP	780/60 BP	PE-Cy7	CCR6	BD Biosciences (560620)	2.5
			655 LP	695/40 BP	PerCP-Cy5-5	FoxP3	ebioscience (45-4776-42)	5
			556 LP	585/42 BP	PE	β7	BD Biosciences (555945)	5
				488/10 BP	SSC			
Red	633	Trigon	735 LP	780/60 BP	APC-H7	CD3	BD Biosciences (641397)	2.5
			690 LP	730/45 BP				
				660/20 BP	APC	CCR9	Biolegend (358908)	5
Violet	407	Octagon	595 LP	610/20 BP				
			502 LP	510/50 BP	AmCyan	Live/Dead fixable dead cell stain	Molecular Probes (L34957)	2.5
				450/40 BP	V450	CD4	BD Biosciences (560345)	2.5

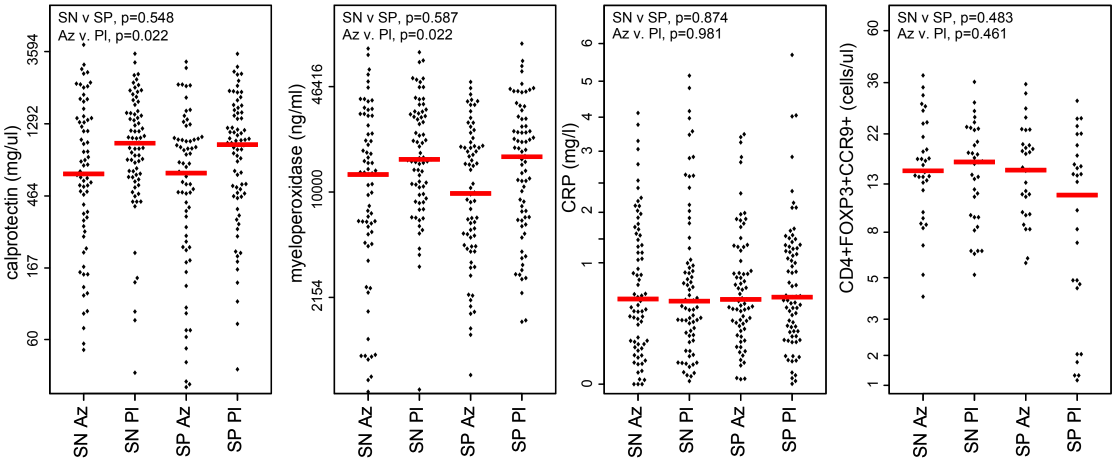

Supplementary Figure 1 Differences in biomarkers of intestinal and systemic inflammation and *ex vivo* regulatory CD4+ T cells according to study arm and seroconversion status. Faecal calprotectin and myeloperoxidase, C-reactive protein (CRP) levels in plasma and the proportion of CD4+ cells in the *ex vivo* flow assay that were CCR9 and FOXP3 positive (CD4+FOXP3+CCR9+) are shown (n=292 infants, except for CD4+ cells n=129). The red bars indicate the median in each group and p-values are shown for the Wilcoxon rank sum test for differences by seroconversion status or study arm after false-discovery rate (FDR) correction (as for Table 1). Abbreviations: SN=seroconversion negative, SP=seroconversion positive, Az=azithromycin arm, Pl=placebo arm. Data are shown on a log-scale with similar values displaced using the beeswarm algorithm so that they are visible.

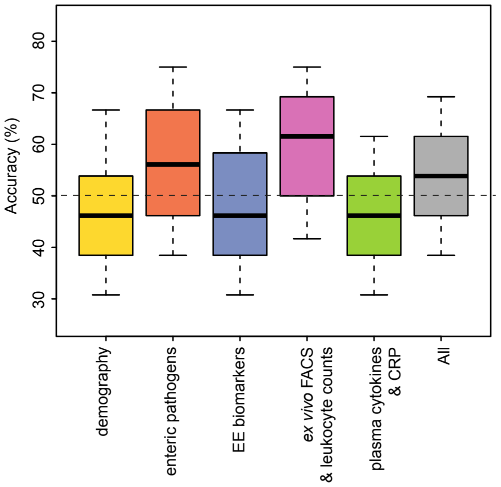

Supplementary Figure 2 Random forests analysis to predict whether shedding of oral poliovirus vaccine on day 7 was detected or not. For each analysis we performed a 10-fold cross-validation repeated 20 times. The boxes correspond to the interquartile range for the accuracies in the prediction set, with the solid line showing the median and the whiskers extending to the 10th and 90th percentile. The dashed line indicates expected accuracy if a random choice were made.

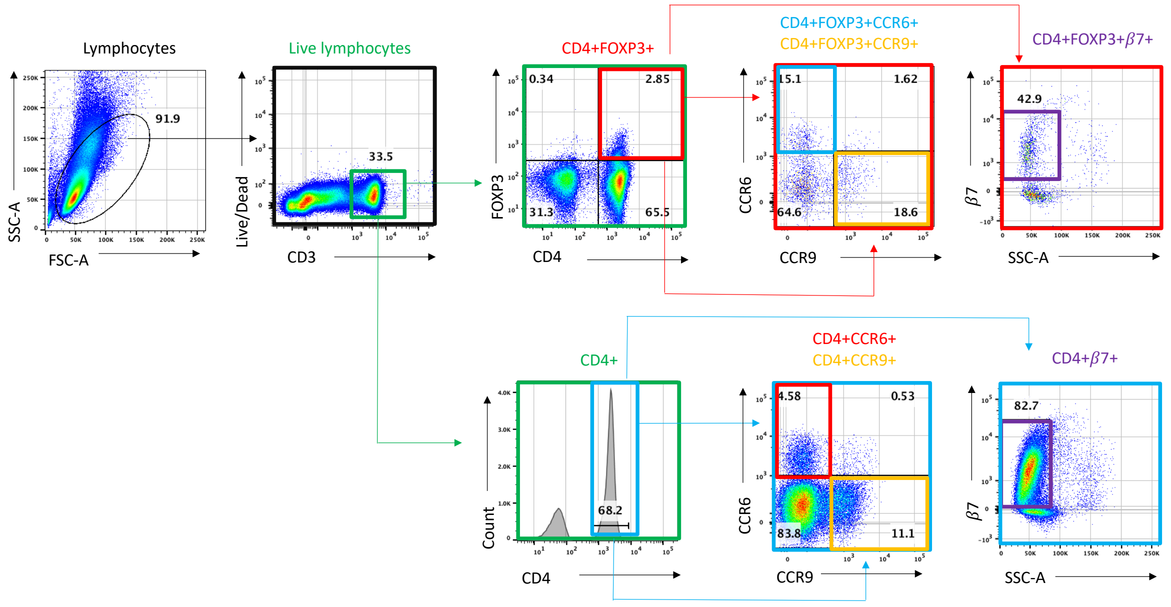

Supplementary Figure 3 Gating strategy for *ex vivo* quantification of lymphocyte populations among peripheral blood mononuclear cells collected at the time of vaccination. A representative set of FACS plots are shown.
